# Teaching comprehensive sexuality education using a praxis co-created with adolescents

**DOI:** 10.4102/phcfm.v15i1.3855

**Published:** 2023-04-17

**Authors:** Ronél Koch, Christa Beyers

**Affiliations:** 1 Foundations of Education, Faculty of Education, University of the Free State, Bloemfontein, South Africa

**Keywords:** adolescent sexuality, comprehensive sexuality education, Life Orientation, praxis, qualitative, South Africa

## Abstract

**Background:**

Despite its reported positive deliverables, comprehensive sexuality education (CSE) in South African schools is unable to document an influence in reducing alarming statistics regarding adolescent sexual health. Prior research points to a gap that exists between what studies suggest and what is implemented in practice.

**Aim:**

Drawing on Freire’s theory of praxis, the aim of this study was to involve the voice of adolescents in reforming CSE – specifically, how the programme could be developed with the objective to co-construct a praxis in order to support sexuality educators in a delivery of CSE that is more responsive to the needs of adolescents.

**Setting:**

Ten participants were purposively selected from all five school quintiles in the Western Cape province of South Africa to take part in this study.

**Methods:**

A qualitative descriptive design with aspects of a phenomenological approach was utilised. Rich data were collected by means of semistructured interviews and were analysed thematically with ATLAS.ti.

**Results:**

The results illustrate the suggestions made by the participants towards the improvement of the CSE programme. They reported on approaches and strategies used to teach CSE that imply that it is often not delivered comprehensively – confirming the disjuncture between what the curriculum envisages and what is executed in practice.

**Conclusion:**

The contribution might lead to change in disconcerting statistics and consequently an improvement in the sexual and reproductive health of adolescents.

**Contribution:**

The participants from this study assisted in co-constructing a praxis for CSE teachers to inform their practice.

## Introduction

Comprehensive sexuality education (CSE) has been part of the South African school curriculum – more specifically, within the subject Life Orientation (LO) – since 2000. However, in all that time it has not succeeded in impacting the sexual behaviour of learners to such an extent that significant changes were evident in alarming statistics regarding the sexual health of young people in South Africa.^1^ Even after a review of the programme and the implementation of scripted lesson plans (learner and teacher support materials paired with supporting activities that are designed to aid teachers and learners to address CSE topics in a systematic manner and to improve uniformity and rigour in the delivery of the curriculum),^2,3^ dire statistics are still rife. In 2020, 34 587 babies were born to South African girls aged 17 years and younger, with 688 of those babies having mothers as young as 9 and 10 years old.^4,5^ The various documented positive impacts of exposure to CSE, such as a delayed initiation of sexual intercourse, decreased frequency of sexual intercourse, decreased number of sexual partners, reduced risk-taking, increased use of condoms and increased use of contraception,^6,7^ lead to confusion as to why CSE in South African schools is unable to report similar outcomes.

Since the implementation of CSE, numerous research studies have been conducted that investigated its status in South Africa. A systematic review by Koch and Wehmeyer,^8^ that included 22 such studies, aimed to determine how the programme contributes to the sexual health of adolescents. The study included research over a 17-year timespan and made substantial recommendations towards the improvement of the programme. Key among these recommendations is an increase in the practice of fidelity. However, there is often a decrease in success when neglecting to translate research findings into practice.^9^ Sexual health could be improved if knowledge about what was learned through research is more successfully applied in practice.^10^

To attend to this gap that often exists between theory and practice,^11^ the authors relied on Paulo Freire’s theory about praxis^12^ as a theoretical and philosophical framework to guide and conduct this research.^13^ Freire^12^ defines praxis as reflection and action upon the world in order to transform it. Those who engage in praxis-oriented research involve the group under study in their research process.^13^ The authors were committed to praxis by involving and seeking to understand the perspectives of participants about the effectiveness of CSE and to develop research that arises out of their experiences and concerns.^13^ The research process created potential for the adolescent participants to be changemakers,^13^ not only because they were empowered by being part of the research process but also because they were involved in developing the CSE programme to impact their lives and the lives of others significantly. It helped the adolescents recognise that they have the ability, through praxis, to transform their circumstances.^14^ The authors therefore acknowledge adolescents as beings who, through reflection and action, can transform their social circumstances in progressive ways.^14^

Furthermore, praxis is distinct because its explicit goal is to empower marginalised people and help them challenge their oppression,^13^ giving them the right to participate in all matters affecting them.^15^ Through praxis, the oppression of adolescents is eliminated by pursuing the notion that, contrary to how they were traditionally excluded from participation in research that is about them,^16^ they have now become active in the promotion of models of learning and social interaction that have a fundamental connection to the idea of their emancipation.^14^

Consequently, it was the aim of this study to involve adolescents who are taught CSE in South African schools, to understand from their perspective how the programme could be developed, with the objective to co-construct a praxis to support sexuality educators in a delivery of CSE that is more responsive to the needs of adolescents. The authors aimed to answer the research question: How can the lived experiences of adolescents be used to co-construct a praxis that educators can use to deliver the CSE programme for it to be more responsive to their needs?

## Research methods and design

A descriptive qualitative research design was deemed most appropriate, with aspects of a phenomenological lens of inquiry, because the authors were interested in obtaining insight into the life-worlds^13,17,18,19^ of adolescents, and in-depth subjective accounts^20^ of how they perceive the sexuality education (SE) that is offered to them.^21^

Moreover, the authors were interested in co-constructing a praxis with adolescents – the worldview that was most suitable for this study was an advocacy or participatory one.^22^ This again links to praxis, indicating that adolescents are a historically marginalised group because of being typically excluded as subjects in research that deals with the issues they face.^23^ However, they were given a voice and the opportunity to take action in the creation of an agenda to change and improve their lives.^21^ Kemmis and Wilkinson^24^ explain that this worldview is emancipatory: it helps unshackle people (adolescents) from the constraints of irrational and unjust structures that limit self-development and self-determination, as is often the case because of the way CSE is typically presented. The research process was practical and collaborative, because it was completed *with* adolescents rather than *on* or *to* them. The authors therefore engaged the adolescent participants as active collaborators.^24^

After obtaining ethical clearance from the Faculty of Education Ethics Committee at the University of the Free State, together with permission from the Western Cape Education Department and school principals, possible participants were identified by LO teachers from respective schools. The participants and their parents or guardians signed assent and consent forms, whereafter data were collected at the respective schools by means of semistructured individual interviews. Probing and follow-up questions were posed to enable a comprehensive exploration of the life-worlds of adolescents.

For a qualitative descriptive study with aspects of a phenomenological lens, a smaller unit of analysis is advised.^25^ Ten adolescent participants (five male and five female) from the Further Education and Training phase of schooling were therefore selected from five high schools, one from each school quintile in the Western Cape of South Africa, to ensure that a wider South African context could be represented. In South Africa, all public schools are categorised into five groups, called quintiles, largely for purposes of the allocation of financial resources. Schools in quintile 1, 2 and 3 have been declared no-fee schools, while schools in quintiles 4 and 5 are fee-paying schools.^26,27^ In the sampled group, there were six mixed-race, two Caucasian and two Black participants, speaking Afrikaans and English, ranging between Grades 10–12 (ages between 15 and 18 years). Purposive sampling was therefore used – a type of nonprobability sampling where a particular case is chosen because it illustrates some feature that is of interest for a particular study.^28^

As suggested by Miller and Salkind,^29^ the collected data were analysed and triangulated with the six phases of a thematic analysis.^30,31^ Interviews were conducted, self-transcribed and translated in order to be fully immersed in the process of a data analysis. Trustworthiness, in particular credibility, was ensured during data analysis by utilising two independent coders who developed and cross-checked the initial codes to achieve interrater reliability^32^ and by using the qualitative software program ATLAS.ti (ATLAS.ti Scientific Software Development GmbH, Berlin, Germany). From the interrelated codes,^25^ themes and subthemes were created inductively to ensure that the data were uncontaminated by personal bias.^22^ The data were therefore analysed thematically.

All of the ethical aspects of research, such as voluntary participation, informed consent and assent, the avoidance of harm, no deception, anonymity and confidentiality, no violation of privacy and the debriefing of participants after the completion of the project, were adhered to.^33,34,35^ To protect the identity of participants, ‘P’ is used instead of their names, the sequential order of the interview and the gender (B or G) the participant identifies with, as well as the quintile school (Q) they are from. For example, ‘P1BQ5’ was the first participant who was interviewed and identified as a boy from a quintile five school. Rich descriptions of the participants’ life-worlds were accounted for with interviews ranging between 27 min and 43 min.^21,36,37^

### Ethical considerations

The research process was cleared by the University of the Free State’s General and Human Research Ethics Committee (ref. no. UFS-HSD2021/0024/123).

All procedures performed in this study involving human participants were in accordance with the ethical standards of the institutional and national research committee and with the 1964 Helsinki Declaration and its later amendments or comparable ethical standards. The confidentiality of data is maintained by storing data on a double password-protected device that only the primary author has access to.

## Findings

Seven themes emerged from the data analysis, deriving initially from 58 codes and 12 subcategories. By relying on the lived experiences of adolescent participants, the following recommendations were made by them with the aim to improve the current CSE programme. Quotes were added *verbatim* to corroborate the themes that emerged.

### Cover content comprehensively

The most dominant theme that emerged from the data was that the participants would like teachers to cover all the content as prescribed by the curriculum and to cover it in depth. It was mentioned how the textbook sometimes covers content more extensively than what is in fact dealt with in the classroom:

‘I also read every now and then and through the textbook before I write test and then I will see that the textbook is much more open about it. So, the textbook is not always one-sided. Like in class, with the teacher, then I would just say also have more conversations about it and not always be one-sided about it.’ (P2GQ5)

The participants specified that they view it as being to their disadvantage if teachers decide to exclude certain content from their learning experience. They were of the opinion that if the content were covered comprehensively, they would be better prepared to deal with the challenges that might come their way. They passionately requested that nothing should be kept from them:

‘People have to tell me what’s going on. So, I will listen to anything. If you tell me what is contraception, and what is that, then I will listen. Because why? I need to know this; I need to know everything to adjust in life.’ (P9BQ1)

‘You might end up when you grow up and you maybe fall pregnant and you’re like, no one told me this, I wasn’t taught, I didn’t know! Why didn’t anyone tell me those were the procedures that I could follow in this? So, I feel like the education is very important.’ (P4GQ4)

Even if a variety of topics are covered, the participants were dissatisfied regarding topics that are merely touched on superficially. They would like content to be covered objectively, comprehensively and thoroughly. The participants mentioned how teachers tend to decide what to include and exclude from the curriculum based on their subjective and sometimes moralistic views as to what is appropriate, as well as their level of comfort in discussing the topics with learners. When teachers use this approach, the participants were of the opinion that there is no room for other views, and it discourages participation from learners with varying opinions. Some of the participants felt that they are judged by teachers and peers when asking questions. It is as if their questions are an indication of what is going on in their personal lives, and this further discourages interaction:

‘He [*the teacher*] does not go into detail. He will just touch the surface.’ (P5BQ3)

‘Now the one might be asking a question, then the learners think it’s about that one’s life.’ (P10GQ1)

In general, the participants also shared their opinion that too little time is spent on SE. They argued that such an important topic is deserving of more time. Some believed the topic to be of the utmost importance, stating that it should be a subject in its own right, taught separately from LO:

‘It’s like a chapter or two in your textbook and they also move very quickly over it.’ (P2GQ5)

‘Many learners do not pay attention in LO periods, it’s where people sleep or do homework, so I think maybe if one every now and then like a course like that we had in Grade 6, a course that learners can attend during their grade period or grade assembly meeting because then one also pay more attention than in LO.’ (P2GQ5)

‘I think, instead of making the sex ed part of LO, it should be made his own [*a separate*] subject.’ (P3BQ4)

### Learning should be interactive, cooperative and participatory

The second biggest theme that emerged was that teachers still tend to rely on traditional methods of teaching where learners are passive onlookers in the process of learning. The participants voiced their dissatisfaction with such methods, stating that they prefer to be more actively part of lessons:

‘Do you know there are a lot of teachers I’ve heard of already, who uhm, they do not talk to the children, they basically read what’s going on in the book.’ (P5BQ3)

‘Last year, we had to copy the textbook, the whole textbook … it does not benefit the children to write everything down, Miss. They get nothing out of it, not even examples.’ (P6GQ3)

‘Sometimes it’s just words and words and words and words, and read and read and read, which a person doesn’t actually absorb.’ (P4GQ4)

‘Teacher has not yet asked us questions about what we think about sex or so, but he is with the book. He will open his notes and then he will read to us what sex is about, he will read it to us more, but he will not ask questions.’ (P10GQ1)

One of the participants mentioned specifically how a teacher used two hard-boiled eggs to explain sexual promiscuity. She smashed one egg, comparing it to a girl who is sexually active, and the other egg without any damage to a girl who preserves her virginity. The participant mentioned how she recently got a boyfriend, and how she still thinks she would like to be a ‘good egg’ and not a ‘bad egg’, but she lacked the skills on how to say ‘no’ to the pressure of wanting to engage in sexual activity with her boyfriend. The participants would like the learning experience to be one where skills could be practised:

‘I do not know, like when you’re in a relationship, like the temptation is always there and I think they do not always teach you, you are always going to sit with it, and it’s hard and yeah, like for example, I got a boyfriend like two months back and it’s always sitting there, but it’s like no I can’t, I have to wait. And they do not teach you that.’ (P2GQ5)

The participants would also like lessons to be more interactive and hands-on, where they are allowed to reflect upon their own realities:

‘I want them to show me, for example, sorry if this is inappropriate … They put something here, then they say this is how you put [*on*] a condom. This is how you do this. That will be better, more face to face.’ (P3BQ4)

‘You can also ask them what is happening in their community, especially now like teenage pregnancy … I believe it happens in every community. You can ask them questions about their community and so on. As what is happening in reality around them and how can they improve it.’ (P10GQ1)

The participants, in fact, praised teachers who make use of interactive methods to teach SE:

‘I feel like my teacher personally, she’s really good because she really gets to interact with the learners. She actually connects with the children, like, tells them, like, yeah. It’s a more intimate conversation with each and every one of the learners in the classroom.’ (P4GQ4)

### Comprehensive sexuality education is imperative and must be implemented in all schools

The participants revealed that their exposure to SE through LO has been limited or once-off – for some even nonexistent – and that most of their exposure to SE has been through outside organisations who visit the school for the purpose of educating children mainly about the prevention of harmful consequences of having sex. They shared the belief that teachers sometimes think it is better for learners to receive no information at all, as the sharing of information will put ideas in their heads that will cause them to think and do things that they would otherwise not have considered. The participants were of the opinion that learners who are excluded from SE are at a disadvantage, because they will not be prepared for the choices they will have to make. They emphasised the importance of SE, and that it should be a compulsory subject:

‘So, I don’t know why the teachers don’t do it but I think it would be amazing if they were forced to teach us, not to choose. It should be mandatory.’ (P3BQ4)

‘I cannot really remember in Grade 11 that we did anything about sexuality education at all, but I do not know if they just took it out due to COVID, but I cannot remember anything about it at all. But in Grade 10 it was also very quick. In high school we do very little about it, but sexuality education I do not think I have heard any of it from school.’ (P2GQ5)

‘In Grade 6 people came to our school and talked to us about it. They showed us photos of what the stuff looks like. I think it’s one of the biggest encouragers not to be sexually active [*he laughs*]. But it’s mostly that and contraceptives, like condoms and so on, that I think was the main focus that has been talked about, right, in this one period, and not really touched on otherwise again.’ (P1BQ5)

‘So, they talked about it once-off?’ (Interviewer)

‘Yes, today is the [*he uses his hands to make quotes*] “sex lesson”.’ (P1BQ5)

‘No, in LO we mostly only deal with stress management. Not really sex education.’ (P10GQ1)

### Use more positive approaches

In contrast to the preference of participants for teachers to deal with content in the above-mentioned manner, the participants revealed that in general, teachers tend to focus mostly on the harmful effects of sexual activity, and they neglect and omit other content:

‘We have very few LO lessons, but it’s more like, okay yeah, uhm, this is sex, don’t do it. Next topic.’ (P2GQ5)

Some of the tactics used by teachers are so shocking that the participants could remember it years after being exposed to it, such as being shown graphic images of sexually transmitted infections. The participants believed that teachers use such tactics to try and scare them away from sex:

‘It almost scares you away from sex. Uhm, but that is also unhealthy, because it does not balance out by teaching you that your sexuality is okay and healthy and normal. If it makes sense?’ (P1BQ5)

Although the participants acknowledge the importance of learning about contraception and the prevention of diseases and infections, they find this one-sided approach harmful and would appreciate a more objective approach where the personal beliefs of teachers are not forced onto learners and where positive aspects of sex and sexuality are included:

‘I think the off-putting aspect, of how sexuality is not portrayed as a healthy, normal part of you, because I also think with teenagers it’s a big problem. We are all terribly uncomfortable with our bodies and who we are. Uhm, and … [*sigh*]. The fact that it’s almost like the danger and thing you have to be careful of and have to, uhm, almost try to stay away from. They say abstinence is the best way and that’s true, but that’s the way sexuality is portrayed. I think is damaging. I think portraying sexuality as a bad thing is damaging to children, it’s bad for your self-esteem, and I think everyone has sexual urges when they’re young. And when I hit puberty, my reaction to that was shame.’ (P1BQ5)

### Promote egalitarian gender norms

The participants often mentioned, intentionally and unintentionally, how different gender roles, gender biases and gender discrimination are at play and reinforced in CSE. They indicated that specifically girls and women who engage in sexual activity lose their worth because of their actions. One of the boy participants shared the example he heard from a teacher where a woman who had more than one sexual partner was compared with a gift that was opened and then regifted, losing her value after each regift. More examples were shared that place an emphasis on how particularly girls and women are judged for being sexually active:

‘I think they should talk more about how the girls should protect themselves from every boy out there and they should say no … the time is not right now. And who is looking for a wife now after a few years, that he thinks it is the woman I want to marry, and then you find out that she’s been lying with so many boys already, and stuff … What do you have left for yourself then.’ (P7BQ2)

‘You feel you will lose some respect for her?’ (Interviewer)

‘Yes.’ (P7BQ2)

‘Now your boyfriend leaves you, you get another boyfriend, and so on … then people are going to classify you as bad, because you’re going from boyfriend to boyfriend. So many failures will happen. So, they’re also going to classify you as bad, and I think mostly one can wait until you are married.’ (P10GQ1)

In addition, the onus seems to lie specifically on girls, rather than on both genders, to abstain from sex and take responsibility for its consequences:

‘I think it’s because if they managed to get this message in the girls’ heads. If the girls can decide, no we’re going to abstain, then the boys won’t have anyone to actually have sex with. If they get one party on the right path, then, yeah.’ (P4GQ4)

The male participants, however, felt frustrated. They are often viewed as scapegoats and blamed for things that go wrong in sexual relationships:

‘What I like the least is when they keep blaming the boys over the matter. It’s always the boys’ fault. But what my mother told me is that the girl always has the last say about her body. So, it depends on both.’ (P7BQ2)

The SE of boys is specifically deemed to be neglected, and the advice provided by the participants is that equal attention should be given to both genders:

‘I feel most of the time when they speak about sexuality, they speak more to girls than boys.’ (P4GQ4)

‘Ever since primary school it was always the girls, because they would be given their stuff for their periods and everything, but we were never taught anything.’ (P3BQ4)

### Learn from real-life examples

The participants highlighted how they benefit from learning from other people’s experiences, especially from people who have made mistakes in the past. They welcome case studies or class visits from individuals who share their insights with them:

‘People have to just volunteer themselves to say I will talk about my experience with it and so on. Schools should get this. Just get people to come talk. This will help a lot. For example, if someone went through the same experience, then they will just feel but look where that one actually is and see where I am now, I also have a younger baby, so I can rise above my circumstances and so on.’ (P8GQ2)

‘Maybe if we show you a bit real life consequences, they will be able to understand why it should not be done.’ (P3BQ4)

‘Do you mean like a video of someone who talks about, or maybe a visitor?’ (Interviewer)

‘Yes, someone who actually went through it.’ (P3BQ4)

‘I think people talk to kids about this but only to inspire them too about what they went through, and not to lead them on the same path they went through. To keep them on the right path.’ (P7BQ2)

‘Or like examples of people who ended up in the situation and how they dealt with it so you more or less have an idea of how you can deal with it.’ (P2GQ5)

## Discussion

The results of this study indicate that based on the lived experiences of the participants as receivers of CSE, they are aware of the misalignment between CSE and their lived experiences. The participants would like content to be covered comprehensively concerning the amount of time spent on it, as well as the depth in which they are allowed to delve into topics, and no content should be omitted from what is discussed with them in class. They prefer interactive learning where they can actively participate in the process of learning. It should be highlighted that although the participants deemed it important to know about the consequences of irresponsible sexual activity, a more positive approach should be employed when teaching about sexuality, such as intimate relationships, desire and pleasure, without causing shame for wanting to explore such aspects. The participants proposed that egalitarian gender norms should be advocated, rather than gender biases and discrimination that are often projected onto them based on the subjective views of teachers. Lastly, the participants favoured learning from real-life examples through case studies shared in the textbook or class visitors who share their experiences.

It is ironic that most of the issues highlighted by the participants are already supposed to be part of practice, as CSE forms part of a compulsory, well-researched and developed curriculum^1^ that is currently implemented. The results concur with prior research reporting on reasons why CSE is often not correctly implemented, including a lack of teacher training, teacher discomfort, authoritarian teaching methods that exclude learners from a solid learning experience, the omission of content to protect the innocence of learners, the use of one-sided approaches (such as abstinence only) or the use of consequences as scaring tactics and the disregard of pleasure and fulfilment.^8^

The participants identified the problem with CSE delivery to be twofold: (1) SE content delivered is not comprehensive enough, meaning that only certain topics (mostly pertaining to the prevention of harmful consequences of sex) are attended to as decided subjectively by teachers, and (2) topics are not taught comprehensively, which means teachers only touch the surface of topics that they do in fact teach about, but they do not allow for in-depth discovery, reflection and discussion. Some of the approaches followed by teachers are not responsive to the needs of adolescents and could even be viewed as damaging, as ignorance will hamper adolescents in their ability to protect themselves from harmful practices or sexual exploitation.^6,23,38^

The themes that emerged from this study indicate a unified, categorical appeal from learners to teachers to change their teaching approaches firstly by making learners a more active part of SE lessons. This request is supported by Kolb’s^39^ experiential learning theory, also known as active learning, interactive learning, or learning by doing that has resulted in many positive outcomes.^40^ Active learning provides: (1) learners the opportunity to build confidence, and it encourages self-learning; (2) ready directions for the application of skills that learners might learn from CSE; (3) teachers with directions for the necessary range of education methods; (4) effective connection between theory and practice; (5) learners with the means to reflect on and provide feedback in order to stimulate their learning about sexuality; and (6) an opportunity to rationalise the way of combining different learning styles that might appeal to different learners so that learning can become more effective,^41,42^ all of which could be to the benefit of teachers when teaching about sexuality and learners when learning how to act assertively when making sexual decisions.

Secondly, the participants implore that more positive approaches be used when teaching about sexuality. Research confirms that CSE is predominantly taught from a discourse of danger and disease,^8,43,44,45,46^ with an emphasis on the consequences of sex and where abstinence and innocence are promoted. The implications thereof are that adolescents are inadvertently prevented from getting access to necessary information and services related to sex.^43^ It also ‘gives rise to presumptions of gendered responsibility for risk and the requirement of female restraint in the face of the assertion of masculine desire and predation’.^46^

Thirdly, the promotion of egalitarian gender norms in the SE classroom relates to another appeal made by the participants. The United Nations Educational, Scientific and Cultural Organization (UNESCO)^6^ informs that gender-focused programmes are known to be significantly more successful than ‘gender-blind’ programmes at reaching health outcomes such as reducing rates of unintended pregnancies or sexually transmitted infections. The inclusion of content that is transformative, as well as teaching methods that support learners to question social and cultural norms around gender and to develop gender-equitable attitudes, are desperately needed in classrooms.^47^

A study by McRee^48^ discovered that teachers who include guest speakers covered more sexuality topics and were more likely to cover controversial topics – therefore increasing the comprehensiveness of sexuality content. Perhaps that might be related to the reason why, fourthly, the participants promote learning from guest speakers and real-life events that they bring to classrooms. Learners tend to view guest speakers as ‘experts’,^49^ and are often more comfortable discussing sensitive topics with someone other than their classroom teacher.^50^ This finding is consistent with comments made by the participants that show that the teaching methods of teachers are sometimes lacking. Teachers should embrace learning opportunities and strive to meet the needs of learners as guest speakers often do.

This discussion leaves one to draw the inference that what adolescents are asking for is already well reported but that a change in practice is necessary. In 2010, it was reported that the CSE programme was not being implemented uniformly.^51^ Today, it is not the curriculum that is lacking but rather the fidelity of the delivery of CSE that varies to such an extent that SE content is sometimes to the detriment of learners’ education. With a sound curriculum in place, a paradigm shift regarding the pedagogical practice of teachers is needed – they are encouraged to engage in self-reflexivity where they acknowledge their own prejudices and identify their values and beliefs as a separate entity from the content that they teach.^52,53,54^ A ‘paradigm shift’ was a term coined by Kuhn.^55^ A fundamental change is needed in the basic concepts and experimental practices of a scientific discipline: teachers need to adapt their CSE teaching practices.

What could devise self-reflexivity and a paradigm shift is a praxis in the form of a workbook for CSE teachers that is co-constructed with the participants from this study, aimed to attend to matters that obscure the successful implementation of CSE. Such a workbook could be used by tertiary institutions for the training of preservice CSE teachers and by the Department of Basic Education (DBE) when they perform in-service training with CSE teachers. Student and practising teachers are then prepared to be responsive to varying contextual demands, ethical imperatives, the diverse learning needs of learners, new modes of delivery and specific professional expectations and transformation priorities when training and teaching CSE. A recommendation for future research is to develop a workbook as such and to make use of preservice and in-service teachers as participants to use the workbook and to afterwards reflect upon their experience in a focus group discussion to make recommendations for theory-building^11^ towards the improvement and implementation of CSE in practice.

The strength of the study lies in the fact that the oppression of adolescents as a previously marginalised group was eliminated and that recommendations for a praxis were created to improve the CSE programme taught in South African schools, so that research recommendations that often do not go beyond that level could be transferred to practice. Limitations could be that a larger sample size could possibly deliver more suggestions with increased meaning saturation,^56^ and that the male participants might have shared different insights with an interviewer who was also male because of possible discomfort of sharing insights regarding their sexuality with a member of the opposite sex. Even though some of the participants from this study might belong to the LGBTQI+ (or those questioning their gender identity or sexual orientation), intersex and other group, the study did not focus per se on whether learners belonging to this community approve or might have other input to include.

## Conclusion

To respond to the troublesome state of the sexual health of adolescents in South Africa, it was necessary to investigate why the CSE programme in South African schools is not yet capable of making a significant difference. Results from this study and other studies prove that valuable recommendations were made towards the improvement and development of the programme but that a gap still exists between research and practice (knowing in theory what needs to change, but neglecting to put this into action). In this research study, the participants were adolescents who were typically historically excluded from research that is about them. They took action by participating in research that was about them, as informed by Paulo Freire’s theory. Their lived experiences were reflected upon to form the foundation of a co-constructed praxis for sexuality educators. The findings could establish fidelity in the delivery of the CSE curriculum and the implementation of scripted lesson plans to ensure that SE in South African schools is in fact comprehensively taught and successful in contributing positively to the sexual health of adolescents in this country.
